# Psychometric Properties of the Persian Version of the College Academic Perfectionism Scale (CAPS)

**DOI:** 10.1002/brb3.70368

**Published:** 2025-03-26

**Authors:** Faezeh Peimanpak, Simin Hosseinian, Abbas Abdollahi

**Affiliations:** ^1^ Department of Counseling, Faculty of Education and Psychology Alzahra University Tehran Iran

**Keywords:** academic perfectionism, perfectionism, psychometrics, sudent, reliability, validity

## Abstract

**Introduction:**

The College Academic Perfectionism Scale (CAPS) is a self‐report 24‐item questionnaire measuring perfectionism in academic and educational environments. The present study determined the psychometric properties of the CAPS among an Iranian sample.

**Methods:**

The statistical sample consisted of students, 404 (115 females and 289 males) of whom were selected by nonrandomized convenience sampling to fill out the questionnaires.

**Result:**

Composite reliability and Cronbach alpha coefficient were used to examine the reliability and internal consistency of the CAPS, and the results revealed the appropriate internal consistency and composite reliability of the scale. Likewise, the face and content validities of the CAPS were evaluated and confirmed. Confirmatory factor analysis was also employed to determine and confirm construct and convergent validities. The analysis of the correlation showed that the CAPS positively and significantly correlated with self‐efficacy, self‐criticism, and perceived stress.

**Conclusion:**

Generally, the results displayed that this scale enjoyed desirable psychometric properties in an Iranian sample and could be applied to Iranian samples confidently.

## Graphical abstract

This study investigated the psychometric properties of the College Academic Perfectionism Scale (CAPS) in an Iranian student sample. Results demonstrate acceptable reliability, validity, and significant correlations with relevant constructs, suggesting that the CAPS is a suitable tool for assessing academic perfectionism among Iranian students.

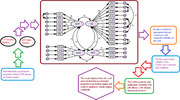

## 1 Introduction

Perfectionism is defined as a personality trait characterized by various properties, such as high self‐criticism (SC) and efforts for perfection (Smith et al. 2022). Perfectionism manifests when individuals set high criteria for their behavior and performance (Vatterott 2019). Perfectionism is a unidimensional trait that leads to negative psychological and pathological outcomes, like a sense of failure, self‐blame, reduced self‐esteem, and depression (Burns 1980). Likewise, others believe it is a multidimensional feature encompassing both positive and negative aspects (Stricker et al. 2020). While positive (normal) perfectionism follows realistic standards, that is perfectionistic efforts, and attains self‐satisfaction and self‐esteem, negative (neurotic) perfectionism involves setting excessively high standards, that is maladaptive perfectionistic concerns (Fang and Liu 2022). Terry‐Short et al. (1995) differentiated between positive and negative perfectionism according to the categorization of normal‐neurotic perfectionism within a theoretical framework. Positive perfectionism refers to a group of cognitions and behaviors that seek to attain high‐level successes and achievements to obtain positive outcomes, while negative perfectionism addresses those cognitions and behaviors that aim to acquire high‐level successes and improvements to avoid or escape from negative outcomes (Terry‐Short et al. 1995).

Indeed, negative perfectionism is a permanent source of stress since individuals continuously oblige themselves to be perfect. Hence, this expectation influences how they cope with stressful situations and often bestows them disappointment (Patterson et al. 2021). Researchers have shown that these two dimensions of perfectionism interact, that is the positive or negative outcomes of perfectionistic efforts depend on individuals' level of perfectionistic concerns (Lv et al. 2023). This result confirms the significance of the multidimensional approach to studying perfectionism and identifying its potential negative consequences. Maladaptive perfectionism is linked to several negative mental outcomes, such as depression (Smith et al. 2021), anxiety (Liu and Berzenski 2022), and low self‐esteem (Shin et al. 2023).

In addition, considering domains wherein individuals manifest perfectionistic traits is paramount. In particular, academic environments may be threatening due to the development of negative and maladaptive perfectionism in these situations. Maladaptive perfectionism is associated with concerns about making mistakes, doubts about actions (DAAs), fear of others' judgments, fear of others' disapproval, and incoordination between expectations and outcomes (Cabaços et al. 2023). This dimension positively correlates with maladaptive indices such as negative emotions. Negatively perfectionistic individuals set high, complex, and unrealistic standards and are occupied with all‐or‐nothing thinking when assessing their performance. Hence, in their mindsets, success occurs when they achieve a criterion within which performance is perfect (Huang et al. 2023).

Thus, scientifically perceiving maladaptive perfectionism and developing accurate tools for this construct is of high clinical and educational significance. Yet, studies have evaluated perfectionism as a personality trait and have not examined this property as a domain‐specific construct, that is a behavior typical of a situation or an environment. However, perfectionism may be specific to a unique domain, for example, education. Studies on perfectionism and other constructs have revealed that special‐domain assessments can predict results more accurately than general scales (Busseri and Mise 2020). For example, Dunn et al. (2005) investigated perfectionism among athlete and nonathlete students and found that athletes manifested higher levels of perfectionism than other students. This finding indicates that perfectionism levels fluctuate in different situations (Dunn, Gotwals, and Dunn 2005). Accordingly, identifying and developing various tools to measure perfectionism in specific domains seems imperative. This is while no reliable instruments exist in the Iranian context for measuring perfectionism in academic environments. One of the international tools in this domain is College Academic Perfectionism Scale (CAPS), developed by Liu and Berzenski (2022). It examines academic perfectionism with 27 items scored on a 6‐point Likert scale, with “1” indicating “Strongly Disagree” and “6” indicating “Strongly Agree.” The CAPS has two higher‐order factors, self‐oriented academic perfectionism and self‐critical academic perfectionism, and self‐critical academic perfectionism consists of three sub‐factors, academic SC, DAAs, and socially prescribed academic perfectionism.

Investigating the psychometric properties of this scale in other societies will result in its widespread utilization. Given that there are very few existing domain‐specific perfectionism measurements, particularly academic perfectionism, and the fact that these measurements are not based on the latest conceptualization of perfectionism, the goal of the current study was to validate the CAPS and assess its psychometric properties in the Iranian students.

## 2 Materials and Methods

### 2.1 Participants

The age range was 15–18, and the data collection method was nonrandomized convenience sampling, so 404 people from secondary schools in Tehran responded to the electronic version of the questionnaire in the Porsline. Females and males constituted 115 (28.8%) and 289 (71.5%) of the sample, with an aged mean and standard deviation of 16.24 ± 0.97. All participants presented their informed consent before completing the online 10‐min questionnaire developed on the Porsline website. This scale collected participants' demographic information, including age, gender, and year of entrance to university, in the December 2023–Feburary 2024 interval according to the test administration guidelines of the Research Ethics Committee of Alzahra University (IR2024‐01‐20).

### 2.2 Instruments

#### 2.2.1 College Academic Perfectionism Scale

Developed by Liu and Berzenski (2022), CAPS possesses 27 items scored on a 6‐point Likert scale, ranging from 1 (*strongly disagree*) to 6 (*strongly agree*), and examines academic perfectionism. With four subscales of Self‐Oriented Perfectionism (SOP), SC, DAA, and Socially Prescribed Perfectionism (SPP), CAPS presents acceptable reliability and internal consistency and enjoys appropriate validity and reliability (Liu and Berzenski 2022).

#### 2.2.2 Generalized Self‐Efficacy

This scale, designed by Schwarzer and Jerusalem (1995), was initially a 20‐item questionnaire with two general and social self‐efficacy subscales. However, it was shortened to a 10‐item scale translated into 28 languages until now. This multiple‐choice 10‐item scale with a 1–4 score range has a minimum and maximum score of 10 and 40 and can predict behavioral changes (Schwarzer and Jerusalem 1995). Farnia et al. (2020) besides confirming a general self‐efficacy (GSE) factor for this scale, confirmed its reliability via the self‐awareness scale and obtained its Cronbach alpha at 0.94. The present research also estimated the Cronbach alpha coefficient and obtained the reliability of this scale at 0.91.

#### 2.2.3 Levels of Self‐Criticism (LOSC)

This scale developed by Thompson and Zuroff (2004) measures two levels of comparative and internalized SC. It includes 22 items scored on a 7‐point Likert scale, ranging from 1 (*strongly disagree*) to 7 (*strongly agree*). Yamaguchi et al. (2022) reported an acceptable internal consistency for this scale (*α* = 0.90) and estimated Cronbach alpha of 0.84 and 0.88 for the comparative and internalized subscales of SC, respectively. The correlation of these subscales equaled −0.66 and −0.52 with self‐value and 0.60 and 0.54 with the emotional instability subscale of the NEO test. In Iran, Mousavi et al. (2014) reported a desirable validity for this questionnaire and estimated a reliability of 0.87 and 0.55 for the comparative and internalized subscales of SC based on the Cronbach alpha coefficient.

#### 2.2.4 Perceived Stress Scale (PSS)

Cohen et al. 1983) built 24‐item scale PSS, every item of which is answered based on a 5‐point Likert scale of *never* (0), *almost never* (1), *sometimes* (2), *fairly often* (3), and *very often* (4). It measures two subscales: (a) negative perceptions of stress and positive perceptions of stress. PSS is applied when we intend to know to what extent individuals perceive their living situations as stressful. The internal consistency reliability of PSS has been estimated at 0.84–0.86 by the Cronbach alpha coefficient among two student groups and one group of smokers in a cessation plan. PSS significantly correlates with life events, depressive and somatic symptoms, utilization of health services, social anxiety, and low life satisfaction (Cohen et al. 1983). In a study conducted by Sharif‐Nia et al. (2024), the results of exploratory factor analysis with Promax rotation extracted two factors accounting for 83.82% of the variance. After necessary modifications during Confirmatory Factor Analysis (CFA), the final model was approved. As for reliability, the Cronbach's alpha, McDobald's omega, CR, and MaxR for all constructs were greater than 0.7, demonstrating good internal consistency and construct reliability.

### 2.3 Procedure

Brislin's method was used to translate CAPS into Persian. First, the original version of SIS was translated from English into Persian by a psychologist professional translators. This was followed by the back‐translation of the synthesized Persian version into English by an English specialist with no knowledge of the English scale and its sentences. Third, the back‐translated version was compared with the original version of CAPS. This comparison was discussed by a committee of three experts from departments of psychiatry, psychology, public health, and clinicians. Any discrepancies found were discussed and changed by the committee. Finally, to receive feedback from participants to check their perceptions of the items and eliminate likely deficits, the researchers distributed the scale among 20 individuals conveniently and removed the present lexical ambiguities (Jones et al. 2001).

Likewise, the SPSS 26 and AMOS 23 software were used to analyze the data and examine the psychometric properties of CAPS.

A qualitative approach was used to examine the face validity of CAPS, for example, a group of five experts and faculties was required to determine the difficulty, unfitness, and ambiguity level of sentences and the semantic insufficiency of words. Content validity, examined quantitatively and qualitatively, evaluates how well items cover the purpose of the topic. For the qualitative part, five faculties were required to provide their corrective opinions in a written form after examining the scale items meticulously and considering grammar, proper wording, the significance and placement of questions, and the completion time of the designed instrument. The necessary modifications were applied after specialists' opinions were collected. Then, the researchers used the Content Validity Ratio (CVR) to evaluate content validity quantitatively and ensure the selection of the most significant and correct content and the Content Validity Index (CVI) to ensure that the items were designed appropriately for measuring the content. For CVR measurement, 15 specialists were required to score every item on a 3‐point Likert scale: *not necessary* (1), *useful but not necessary* (2), and *necessary* (3).

Confirmatory factor analysis with maximum likelihood was used to examine the construct validity of CAPS. Since the factor loading of items should be above 0.4 (Kline 2023), items lacking this feature were excluded from the analysis. Goodness of Fitness (GOF) indices were used to examine the model's validity. For a properly fit model, the Comparative Fit Index (CFI), Tucker–Lewis Index (TLI), and Incremental Fit Index (IFI) should be above 0.90. Likewise, the Parsimony Comparative Fit Index (PCFI) and Parsimony Normed Fit Index (PNFI) should exceed 0.50. Although the Chi‐squared (*χ* ^2^) index is often used to investigate GOF, *χ* ^2^ is related to the large sample size and degree of freedom and, hence, is not usually confirmed. For this reason, the Root Mean Square Error of Approximation (RMSEA) and minimum discrepancy divided by degree of freedom (CMIN/DF) are employed. Thus, if RMSEA is < 0.08 and CMIN/DF is < 3, the model fits acceptably. In CFA, covariation of errors suggests that there are correlations between the residuals of two indicators that are not accounted for by the latent variables in the model. When modification indices suggest covarying errors (e.g., between e1 and e2, e7, and e8), it implies that there are shared sources of variance between these pairs of observed variables beyond what is explained by their respective latent factors. By allowing these error covariances, you are improving the model fit by acknowledging and incorporating these correlations, which leads to a more accurate representation of the relationships between your measured variables and latent constructs (Kline 2015).

The researchers used the Average Variance Extracted (AVE) to examine convergent validity (CV) and Composite Reliability (CR) and utilized the Cronbach alpha coefficient to investigate internal consistency. According to Fornell and Larcker (1981), CR values of > 0.60 indicate acceptable internal consistency of a construct. Besides, AVE values of > 0.50 hint convergent validity.

CR (sometimes called construct reliability) is a measure of internal consistency in scale items, much like Cronbach's alpha. It can be thought of as being equal to the total amount of true score variance relative to the total scale score variance. Cronbach's alpha is a way of assessing reliability by comparing the amount of shared variance, or covariance, among the items making up an instrument to the amount of overall variance. The idea is that if the instrument is reliable, there should be a great deal of covariance among the items relative to the variance.

## 3 Results

### 3.1 Face Validity

The opinions of experts were applied to the questionnaire as slight modifications.

### 3.2 Content Validity

Based on Lawshe's table and considering the number of specialists, if the index value was > 62%, the respective item was necessary and significant at the *p* < 0.05 level (Lawshe 1975). According to the responses, the CVR value of all items fell into the 0.733–0.866 range and equaled an acceptable value of 0.782 for the full CAPS. Likewise, considering simplicity, relevance, and clearness, CVI ranged from 0.866 to 1 for all items and equaled 0.948, 0.945, and 0.914 for the full CAPS, revealing an acceptable level (Waltz and Bausell 1981).

### 3.3 Data Analysis

Before data analysis, the skewness and kurtosis indices were used to ensure item normality. Acceptable values equal ±2 for skewness and ±7 for kurtosis (Hair et al. 2014). The results showed that the skewness of the items fell in this range, while the kurtosis value was > 2 for an item. However, kurtosis was normalized through the identification and omission of two data (Table 1).

**TABLE 1:** Descriptive statistics and factor loadings.

Factors	Items	M	SD	Skewness	Kurtosis	Factor loadings
SOP	P1	4.32	1.78	−0.639	−1.04	0.583
	P2	5.55	0.77	−1.90	3.68	0.651
	P3	5.07	1.13	−1.24	1.27	0.762
	P4	5	1.14	−1.09	0.677	0.756
	P5	5	1.23	−1.27	1.12	0.655
	P6	5.48	0.80	−1.53	2.05	0.572
SC	P7	3.99	1.66	−0.400	−1.03	0.657
	P8	3.37	1.12	−0.712	−0.733	0.244
	P9	4.24	1.66	−0.589	−0.895	0.527
	P10	4.35	1.54	−0.636	−0.670	0.568
	P11	4.31	1.57	−0.646	−0.662	0.638
	P12	3.51	1.67	−0.020	−1.24	0.668
	P13	4.29	1.63	−0.640	−0.789	0.767
	P14	3.69	1.64	−0.134	−1.23	0.733
	P19	4.56	1.78	−0.739	−0.660	0.212
	P20	4.04	1.56	−0.399	−0.862	0.750
	P21	3.48	1.59	0.038	−1.06	0.696
	P22	3.31	1.63	0.152	−1.15	0.760
	P23	3.54	1.68	0.011	−1.21	0.813
	P24	4.31	1.29	−0.681	−0.688	0.201
DAA	P15	4.25	1.56	−0.594	−0.736	0.710
	P16	3.63	1.69	−0.092	−1.28	0.746
	P17	4.07	1.63	−0.528	−0.885	0.717
	P18	3.50	1.64	0.037	−1.15	0.587
SPP	P25	4.31	1.43	−0.630	−0.452	0.400
	P26	2.97	1.76	0.458	−1.16	0.865
	P27	3.11	1.76	0.296	−1.26	0.873
SOP		30.41	5.08	−0.953	0.359	—
SC		42.75	12.96	−0.184	−0.655	—
DAA		15.44	5.10	−0.315	−0.616	—
SPP		10.38	4.02	0.268	−0.851	—
CAPS		98.99	21.99	−0.323	−0.320	—

### 3.4 Construct Validity

The results showed that CAPS included four factors: SOP, SC, DAA, and SPP. Covarying errors were introduced based on modification indices to improve model fit. This adjustment reflects a nuanced understanding of the data and strengthens the model's validity by accounting for additional shared variance not captured by the original factor structure alone. Except for three, all items had factor loadings of > 0.4 and were significant (*p* < 0.01). Thus, items 8, 19, and 24 with factor loadings of < 0.4 were not analyzed.

**FIGURE 1:**
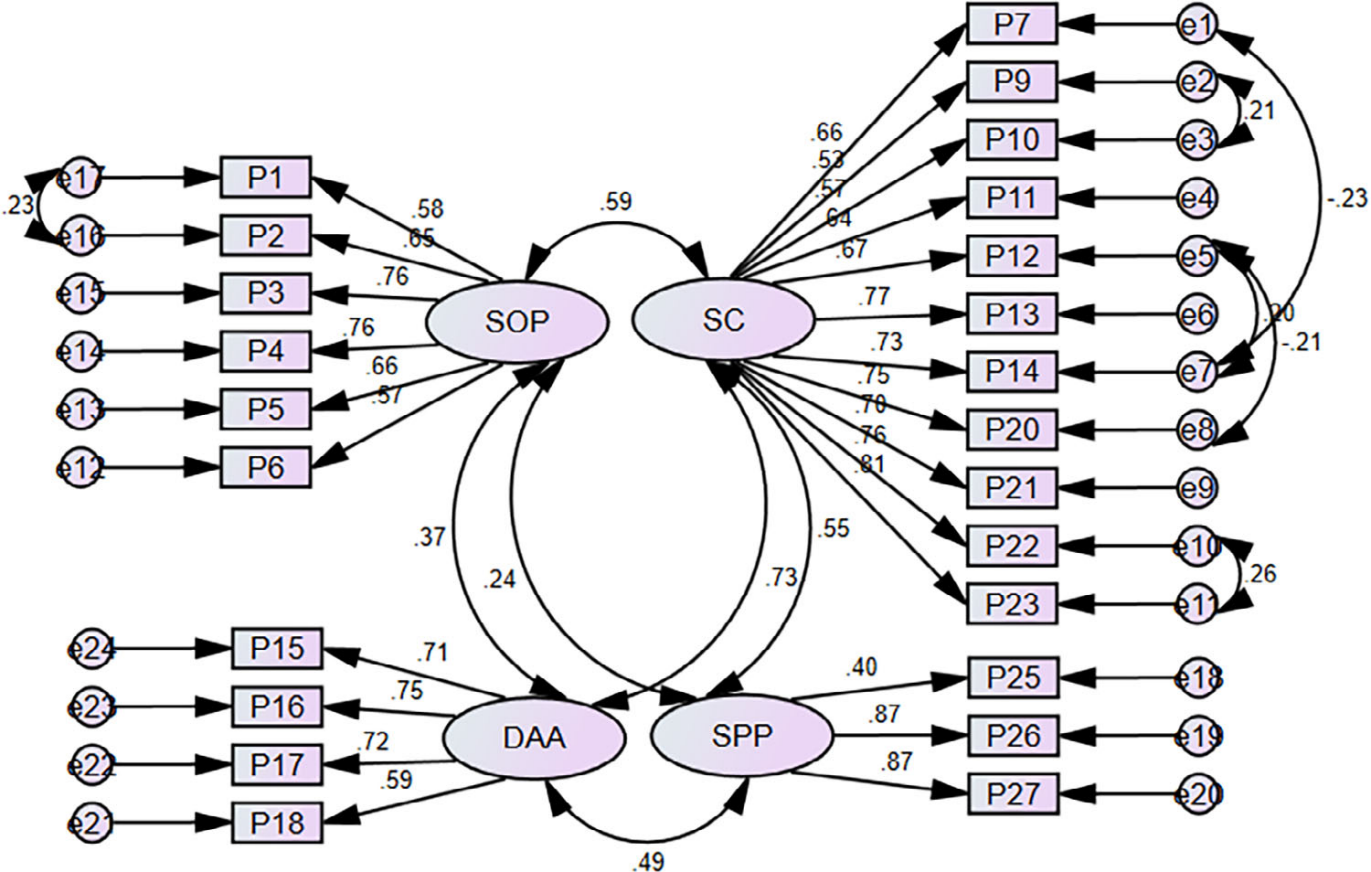
Empirical model of the research.

In the initial model, the fit indices showed that the model was not fit desirably. Hence, the model was modified and reexamined based on the suggested corrections through the establishment of covariance between values. The results displayed that, except for *χ* ^2^, other indices reflected the measurement model's acceptable fit (Table 2). Figure 1 shows the measurement model.

**TABLE 2:** Results of model fit indices.

Index	*χ* ^2^	*p*	*χ* ^2^/df	CFI	RMSEA
Acceptance range	*p* > 0.05		< 3	> 0.9	< 0.08
Result	614.31 (*p* = 0.001)	0.001	2.56	0.917	0.062
	Not confirmed		Confirmed	Confirmed	Confirmed
**Index**	**IFI**	**TLI**	**PNFI**	**PCFI**	**SRMR**
Acceptance range	> 0.9	> 0.9	> 0.5	> 0.5	> 0.8
Result	0.918	0.904	0.758	0.797	0.897
	Confirmed	Confirmed	Confirmed	Confirmed	Confirmed

### 3.5 Convergent Validity and Reliability

The analysis showed that although the total score of CAPS and its factors, except for SPP, did not enjoy suitable convergent validity, they manifested acceptable CR. Furthermore, CAPS with a Cronbach alpha coefficient of 0.921 and its factors with Cronbach coefficients between 0.736 and 0.909 were desirably reliable (Table 3).

**TABLE 3:** AVE, CR, and Cronbach's alpha.

Variable	AVE	CR	Cronbach's alpha	McDonald's omega
SOP	0.446	0.826	0.803	0.81
SC	0.488	0.836	0.909	0.75
DAA	0.480	0.786	0.786	0.74
SPP	0.557	0.775	0.736	0.74
CAPS	0.492	0.805	0.921	0.92

### 3.6 Concurrent Validity

The researchers examined concurrent validity by considering the correlation of CAPS and its factors with the general self‐efficacy, SC, and PSSs. The results showed that CAPS factors positively and significantly correlated with each other, indicating the scale's internal consistency. Moreover, CAPS positively and significantly correlated with self‐efficacy (0.139), SC (0.480), and perceived stress (0.182), revealing the acceptable concurrent validity of CAPS (Table 4).

**TABLE 4:** Correlation between research variables.

Variables		1	2	3	4	5	6	7
1	SOP	1						
2	SC	0.524**	1					
3	DAA	0.263**	0.597**	1				
4	SPP	0.232**	0.561**	0.434**	1			
5	CAPS	0.644**	0.952**	0.724**	0.668**	1		
6	Self‐efficacy	0.271**	0.167**	−0.126*	0.037	0.139**	1	
7	Self‐criticism	0.165**	0.492**	0.432**	0.283**	0.480**	−0.177**	1
8	Perceived stress	−0.123*	0.158**	0.382**	0.158**	0.182**	−0.469**	0.460**

*p < 0.05,**p < 0.01.

**Table:** 

**The Persian version of the College Academic Perfectionism Scale (CAPS)**	
دوست دارم نمره‌های کامل در مدرسه بگیرم. (SOP)	1
برای به دست آوردن نمره کامل در تکالیف مدرسه هر کاری از دستم بر بیاید انجام می‌دهم. (SOP)	2
اولویت‌های من کارهایی هستند که به من در به‌دست آوردن نمره کامل در آزمون کمک کنند. (SOP)	3
نیاز زیادی به گرفتن نمره A در مدرسه دارم. (SOP)	4
تلاش می‌کنم تا حد امکان بهترین عملکردم را در آزمون نشان دهم. (SOP)	5
وقتی در یک آزمون نمره کامل نمی‌گیرم، احترامی که به خودم دارم خدشه‌دار می‌شود. (SWC)	6
اگر نمره کاملی در یکی از تکالیف مدرسه نگیرم، احساس خوبی نخواهم داشت. (SWC)	7
موفقیت تحصیلی هویت مرا تشکیل می‌دهد. (SWC)	8
اگر برای رسیدن به حد عالی تلاش نکنم، نمی‌توانم به خودم احترام بگذارم (SWC)	9
تلاش کردن برای حتی یک تکالیف کوچک ارزش مرا بیشتر می‌کند. (SWC)	10
اگر در آزمون اشتباهی بکنم که نباید می‌کردم، عصبانی می‌شوم. (COM)	11
اگر در یک تکالیف مدرسه اشتباهی بکنم که نباید می‌کردم، شرمسار می‌شوم. (COM)	12
از کاهش نمره‌هایم در تکالیف مدرسه خیلی می‌ترسم. (COM)	13
اگر در یک تکالیف مدرسه اشتباه کوچکی بکنم ناراحت می‌شوم. (COM)	14
تقریباً همیشه بعد از آزمون، درباره‌ی عملکرد خودم شک و تردید دارم. (DAA)	15
اغلب نگرانم که آیا تکلیف مدرسه را به درستی انجام می‌دهم یا خیر. (DAA)	16
نگرانم که آیا من انتظارات استادهایم را برآورده می‌کنم یا خیر. (DAA)	17
درباره کارایی تحصیلی‌ خودم شک دارم، بدون اینکه به عملکردم در کلاس توجه کنم. (DAA)	18
وقتی در آزمونی نمره کامل نمی‌گیرم خودم را به شدت مورد انتقاد قرار می‌دهم. (SC)	19
وقتی استاد اشتباهم را تشخیص می‌دهد از خودم ناراضی می‌شوم. (SC)	20
وقتی عملکردم در یک امتحان عالی و بدون نقص نیست، نمی‌توانم خودم را ببخشم. (SC)	21
وقتی که عملکردم در یک تکلیف مدرسه عالی نباشد، نسیت به خودم عصبانی می‌شوم (SC)	22
اگر نمره‌هایم در مدرسه عالی نباشند والدینم از من راضی نخواهند بود. (SPP)	23
اگر در کلاس‌های اساتید مختلف عملکرد عالی نداشته باشم، از من ناراحت می‌شوند. (SPP)	24
اگر در مدرسه نمرات عالی نگیرم، دوستانم به من نگاه تحقیرآمیزی خواهند داشت. (SPP)	25
اگر در آزمون عملکرد عالی نداشته باشم، همکلاسی‌هایم من را ناکارآمد می‌بینند. (SPP)	26
اطرافیانم انتظار عملکرد عالی از من در مدرسه ندارند. (SPP)	27

## 4 Discussion and Conclusion

The present study investigated the psychometric properties of the CAPS in Iranian society. The obtained results were significant since the researchers examined the statistical properties of this scale for the first time and presented outcomes regarding the intercultural application of this scale.

The face and content validity results indicated that the items of the considered scale were suitable for the Iranian context and were not to be omitted or modified. Confirmatory factor analysis with maximum likelihood was employed to probe construct validity. The results revealed that all items were significant except for three, which were excluded from the analysis (*p* < 0.01). Convergent validity (AVE > 0.50) was also determined and confirmed for this questionnaire. CR and Cronbach's alpha and McDonald's omega were utilized to investigate CAPS reliability and internal consistency. The findings displayed the acceptable CR and internal consistency of this scale. Finally, the analysis of the correlation matrix associated with the research variables showed that CAPS positively and significantly correlated with self‐efficacy, SC, and perceived stress (*p* < 0.01).

In line with these results in the Liu and Berzenski (2022) study, the scale and subscales all exhibited great internal consistency. Convergent validity was established by strong correlations between the CAPS and related and maladaptive constructs (e.g., domain‐general perfectionism, stress, anxiety, depression, and SC). Moderate to weak correlations with the less relevant and nonrelevant constructs (e.g., narcissism, agreeableness, and openness) indicated great divergent construct validity. Interestingly, in the present study self‐efficacy did not correlate strongly with the CAPS as expected, possibly due to the separation between ability and desire/thoughts in perfectionism; in other words, striving for perfection does not equal perceived incompetence.

Indeed, severe self‐analysis, unrealistic expectations, and failure to accept perceived unsuccess cause perfectionists to face problems in accepting past experiences. Furthermore, trying to meet others' expectations makes finding meaningful and valuable experiences impossible for these individuals, and their struggles to strengthen their life stories bring about several outcomes like stress (Liu and Berzenski 2022). This stress can play a significant role in perfectionists' engagement with negligent behavior and is, hence, related to their self‐efficacy. Bandura's (1986) socio‐cognitive theory explains that if a behavior is a function of its outcomes, it can change through cognitive mediators. This theory suggests that our self‐regulation mechanisms can influence the relationship between our emotional arousal and activity or performance. Self‐efficacy is one of the self‐regulation mechanisms. In the socio‐cognitive theory, perceived self‐efficacy plays a central role in provoking anxiety to apply control over potential threats. Individuals who believe they can control these threats are not occupied with distressing and anxious thoughts. Therefore, it is likely that perfectionistic behavior influences individuals' self‐efficacy and performance through anxiety arousal, which, in turn, decreases self‐esteem (Ford et al. 2023).

All in all, the analytical results align with the research findings of Liu and Berzenski (2022), who believed in the suitability of the tool for measuring academic perfectionism, indicating that this scale is desirably valid and reliable and can be used for Iranian samples confidently. In other words, this study presents primary evidence for the acceptable validity and reliability of this scale in the Iranian context. This scale would be useful in both clinical and academic settings; while practitioners can utilize the measurement to identify whether students' mental health issues are related to academic perfectionism and employ targeted therapeutic approaches, the CAPS can also be administered by researchers interested in personality across different disciplines and contribute to the advancement of the scientific understanding of academic perfectionism. Finally, the directional relationships between academic perfectionism (or domain‐general perfectionism) and psychological well‐being have rarely been addressed and should be accounted for with future longitudinal designs.

There are several limitations with the scale. First, participants were recruited from an imbalanced number of males and females (it is worth mentioning that the higher ratio of female to male students may be due to females' greater inclination—especially in higher grades— to participate in surveys and research activities); Although no statistically significant gender differences have been reported. Whether the scale is equally valid in two groups of male and female populations needs further investigation. The second limitation is the estimation of reliability with Cronbach's alpha and McDonald's omega and not the test‐retest method. Also, since the examined samples were from one city, we cannot generalize the results to all individuals in the country. Hence, further studies on more diverse samples are suggested to take more decisive positions about the statistical properties of this scale in the Iranian context. Three items were eliminated from the scale because they either would decrease the reliability of the scale or had cross‐loading issues, resulting in 24 items remaining.

As for convergent and divergent construct validity, the CAPS was expected to be strongly related to the closely related constructs (i.e., self‐efficacy, SC, and perceived stress). It was expected this scale to be moderate to strongly related to these variables because previous research has demonstrated that maladaptive perfectionism is strongly related to cognitions. However, these measures were correlated weakly with academic perfectionism. This is in addition to the importance of studying perfectionism correlation with other personality traits.

## Author Contributions

**Faezeh Peimanpak**: conceptualization, writing–original draft, investigation, funding acquisition, methodology, validation, visualization, writing–review and editing, software, formal analysis, data curation, resources. **Simin Hosseinian**: writing–review and editing, supervision, data curation, conceptualization. **Abbas Abdollahi**: writing–review and editing, supervision, project administration, data curation, methodology.

## Ethics Statement

Informed consent was obtained from all individual participants included in the study.

## Consent

The authors have nothing to report.

## Conflicts of Interest

The authors declare no conflicts of interest.

### Peer Review

The peer review history for this article is available at https://publons.com/publon/10.1002/brb3.70368.

## Data Availability

The data related to the results of this study are available upon request from the corresponding author.
