# Correction to “Psychometric Properties of the Persian Version of the College Academic Perfectionism Scale (CAPS)”

**DOI:** 10.1002/brb3.71160

**Published:** 2026-02-08

Peimanpak, F., Hosseinian, S. and Abdollahi, A. 2025. “Psychometric Properties of the Persian Version of the College Academic Perfectionism Scale (CAPS).” *Brain and Behavior*, **15**. https://doi.org/10.1002/brb3.70368

In the section discussing the Levels of Self‐Criticism (LOSC), the article mistakenly cites the following source:

Yamaguchi S., Y. Kawata, Y. Murofushi, and T. Ota 2022. “The Development and Validation of an Emotional Vulnerability Scale for University Students.” *Front Psychol*, **15**. no. 13: 941250. https://doi.org/10.3389/fpsyg.2022.941250.

However, this paper does not include the LOSC scale or report its reliability. The correct citation should be:

Yamaguchi, A., & Kim, M. S. 2013. “Effects of self‐criticism and its relationship with depression across cultures.” *International Journal of Psychological Studies* **5**, no. 1: 1–1. https://doi.org/10.5539/ijps.v5n1p1

We apologize for this error.

